# The Accumulation of Crocin and Geniposide and Transcripts of Phytoene Synthase during Maturation of *Gardenia jasminoides* Fruit

**DOI:** 10.1155/2013/686351

**Published:** 2013-03-24

**Authors:** Lan Gao, Bi-Yun Zhu

**Affiliations:** School of Basic Courses of Guangdong Pharmaceutical University, Guangzhou Higher Education Mega Center, Guangzhou, Guangdong 510006, China

## Abstract

Gardenia fruit (fruit of *Gardenia jasminoides* Ellis) is used as a natural pigment resource and a Chinese traditional medicine. The white mesocarp turning orange or red that occurs during gardenia fruit maturation arises from the production and accumulation of the apocarotenoids, especially crocin-1, which is derived from carotenoid. Meanwhile, the major medical component geniposide is accumulated in gardenia fruit. To further our understanding of the synthetic and accumulation mechanism for crocin-1 and geniposide in gardenia fruit, the contents of crocin-1 and geniposide and the transcripts of phytoene synthase (GjPSY) profiles in gardenia fruits were examined at various stages of maturation. The concentration of crocin-1 and geniposide in gardenia fruit was determined by reversed-phase high-performance liquid chromatography (HPLC). The results showed that the concentration of crocetin-1 was increased during fruit development and the concentration of geniposide does not change significantly during maturing. The expression levels of GjPSY mRNA were examined by RT-PCR. It was revealed that GjPSY was constitutively expressed during fruit development, suggesting that the primary mechanism that controls crocin accumulation in *G. jasminoides* fruits during development is not correlated to the differential regulation of transcript levels of GjPSY gene.

## 1. Introduction

**Figure 1:**
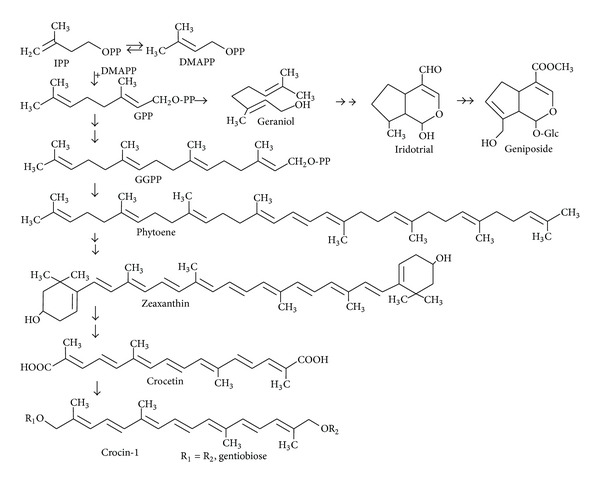
The proposed biosynthesis pathway of crocin and geniposide. Abbreviations: IPP, isopentenyl diphosphate; DMAPP, dimethylallyl diphosphate; GPP, geranyl diphosphate; GGPP, geranylgeranyl pyrophosphate.

Gardenia fruits are widely used in Asian countries as a natural colorant and as a Chinese traditional herbal medicine since they have homeostatic, hepatoprotective, analgesic, antiphlogistic, antipyretic, and hypolipidaemic effect [1]. Geniposide, crocin, and crocetin are the major secondary metabolites in the fruit [2]. Geniposide is a predominant iridoid compound present in gardenia fruits (Figure 1), exhibit a wide range of pharmacological activities, was reported to have hepatoprotective effect [3], hypoglycemic effect [4], insulin resistance-alleviating effect [5], antiproliferation effect [6], antioxidant effect [7], and antioxidant and neuroprotective effect [8], and is a cross-linker to make polymeric material in biomedical applications [9]. Crocetin is a unique apocarotenoid with C_20_ backbone and a carboxyl group at each end; crocin is mono and diglycosyl ester of crocetin; the digentiobiosyl ester compound crocin-1 is the major component of crocin in fruit (Figure 1). The crocin and crocetin derivatives are responsible for the orange or red color of the fruit and are two active ingredients of this herbal medicine. In animal and human studies, it has been shown that crocin and crocetin exhibit a variety of pharmacological effects, such as antioxidant [10], antihyperlipidemia [11], antiatherosclerotic [12], antiinflammatory [13], antiproliferation [14], neuroprotective effects [15], insulin resistance improvement [16], positive effects on sleep [17], and attenuation of physical fatigue [18], and prevent retinal degeneration [19]. Therefore, crocin, crocetin, and geniposide have various pharmacological effects on different illness.

Crocin, crocetin, and geniposide are isoprenoids; they are all derived from C_5_ precursor isopentenyl diphosphate (IPP) and dimethylallyl diphosphate (DMAPP), and the initial steps of their biosynthesis involve condensation of IPP and DMAPP to produce geranyl diphosphate (GPP). From GPP, zeaxanthin is produced through carotenoids synthesis pathway [20]. The formation of phytoene, the first C_40_ intermediate in the carotenoid pathway, has been shown to be rate limiting for the synthesis of carotenoids. Crocetin can be formed through zeaxanthin cleavage [21], and crocin is formed from crocetin by specific glucosylation steps [22]. From GPP, the geniposide is synthesized by monoterpene indole alkaloids (MIAs) pathway; a key step in the biosynthesis is the generation of geraniol from GPP. From geraniol, iridotrial could be produced and then converted to iridoid compounds such as geniposide. Few studies have investigated the biochemical characterization of the iridoid biosynthetic pathway [23]. Though it is known that gardenia fruits naturally accumulate crocin, crocetin, and geniposide, the biochemical pathway leading to these compounds in *G. jasminoides* is not clear; from previous work, we isolated the cDNA coding for phytoene synthase from the gardenia fruit (GjPSY, Genbank accession no. HQ599860). In order to gain insight into the molecular basis of crocin and geniposide synthesis, obtain data concerning major chemicals crocin-1 and geniposide concentration for the determination of optimal time of harvest, and to determine when and if GjPSY transcript levels in the developing fruit correlate with apocarotenoid accumulation, the contents of crocin and geniposide and the transcriptional expression of GjPSY in the fruits at various development stages were analyzed.

## 2. Materials and Methods 

### 2.1. Plant Material

*G. jasminoides* plants cultivated at Guangdong Pharmaceutical University were used as materials. Fruits were collected at 4 development stages from the 8th week to the 36th week after flowering date; the sampling times were the 8th, 16th, 24th, and 36th weeks. Stage I is closed with green exocarp and colorless mesocarp; stage II is closed with yellowish green exocarp and orange mesocarp; stage III is closed with orange exocarp and red mesocarp; stage IV is the the fully ripening stage, closed with half-dried fruit (red exocarp and deep red mesocarp). All samples were identified by one of the authors. The samples were stored at –80°C until used. Methanol-water extract and total RNA were prepared from the fruits and used for HPLC and RT-PCR analysis, respectively.

### 2.2. Chemicals and Reagents

Crocin-1 was purchased from Sigma-Aldrich Corporation (USA); geniposide and authentic gardeniae fruit (powder) were purchased from the National Institutes for Food and Drug Control (China). Acetonitrile (Tianjig, China) was of chromatographic purity, and water was double distilled for HPLC. PrimeScript One Step RT-PCR Kit was purchased from TaKaRa (Japan). All other reagents and solvents were analytically pure and purchased from local firms.

### 2.3. HPLC Analysis of Crocin and Geniposide

The fruits in stages I, II, III, and IV and leaves were analyzed. The 0.1 g fruits and leaves were powdered under liquid nitrogen using a mortar and pestle, and the powdered tissues and the authentic gardeniae fruit powder were extracted with 50% (v/v) methanol water to a final volume of 40 mL. 5 mg crocin-1 was dissolved in methanol to a final volume of 50 mL. 5 mg geniposide was dissolved in methanol to a final volume of 50 mL. The solvents were treated with ultrasonic instrument for 30 min and then were centrifuged at 10,000 g for 10 min at 4°C; the supernatants were collected and stored at −20°C until the HPLC analysis.

For the HPLC analysis, qualitative and quantitative analysis of crocin-1 and geniposide in the samples were conducted as previously described with modifications [24]. The supernatants were filtered with a 0.45 *μ*m filter and were applied to a Diamonsil C18 (2) column (4.6 × 250 mm, 5 *µ*m) from Dikma (Dikma Technologies Inc., China) and then were eluted under gradient conditions using a Waters 2995 HPLC system (Waters, USA) equipped with a 2996 photodiode array detector. Data acquisition and processing were performed using Empower 2 software. The crocin-1 and geniposide were eluted using gradient as follows: 0 to 20 min, 10% acetonitrile in water to 20% acetonitrile; 20 to 25 min, 20% acetonitrile to 50% acetonitrile, at a flow rate of 1.0 mL/min, detected at 440 nm and 238 nm wavelengths for crocin-1 and geniposide, respectively. The peaks were identified by comparing the specific retention time and absorption spectra with crocin-1 and geniposide. The concentrations of crocin-1 and geniposide were determined by the standards curves and expressed as milligrams per gram fresh weight. Crocin-1 and geniposide quantifications were performed in three replicates.

### 2.4. RT-PCR Analysis of GjPSY

Total RNA was extracted from the fruit at stage I to stage III (at stage IV, fruits are half-dried and could not be analyzed), using a modified hexadecyl-trimethyl-ammonium-bromide (CTAB) based extraction protocol with revision [25]. 0.7 g frozen fruits were powdered under liquid nitrogen using a mortar and pestle, transfer the powder into the 65°C CTAB extraction buffer (2% CTAB (w/v), 2% polyvinylpyrrolidone K30 (w/v), 100 mM Tris-HCl (pH8.0), 25 mM sodium-EDTA (pH8.0), 2.0 M NaCl). Just before use, add *β*-mercaptoethanol to a final concentration of 4% (v/v) and shake for 30 min; the mixture was centrifuged at 12,000 g for 10 min at 4°C; the supernatants were collected and extracted with one volume of phenol : chloroform : isoamyl alcohol (25 : 24 : 1). After centrifugation for 20 min, the water phases were removed and mixed with one volume of 4 M LiCl. RNAs were allowed to precipitate overnight at 4°C and collected by centrifugation for 20 min. The pellets were dissolved in 100 *μ*L water; then RNAs were precipitated with 0.1 volume of 3 M NaOAc (pH5.2) and 2 volumes of ethanol at −20°C for 1 hr. After centrifugation, the RNA pellets were washed with 75% ethanol and dried; RNA was dissolved in 50 *μ*L of DEPC-treated water. From the extracted total RNA, cDNA was synthesized using the PrimeScript One Step RT-PCR Kit for RT-PCR. Two degenerate primers were designed for reverse transcription and amplification GjPSY cDNA: forward-5′-GACATACTTGCTGGAAAGGTCAC-3′ and reverse-5′-GCTAGTGGAGATGATGTTCTTGG-3′. The control sample was *G. jasminoides* RPS25-1 gene (Genbank accession no. GU797554); two primers were used for reverse transcription and amplification in the same conditions: forward-5′-CAGAAGAAGAAGAAGTGGAGCAA-3′ and reverse-5′-TGGTAGCTCTGGTGTATATCTGC-3′. The amplification procedure by PCR consisted of an initial denaturing step at 95°C for 2 min, followed by 32 cycles of 30 s at 94°C, 30 s at 55°C, and 1 min at 72°C. The amplified PCR products were analyzed by 1.2% agarose gel electrophoresis. These experiments were repeated in three replicates. To correct the initial mRNA levels, each cDNA band intensity was normalized to the intensity for the RPS25-1 gene amplification.

## 3. Results and Discussion

### 3.1. Crocin-1 and Geniposide Contents

**Figure 2:**
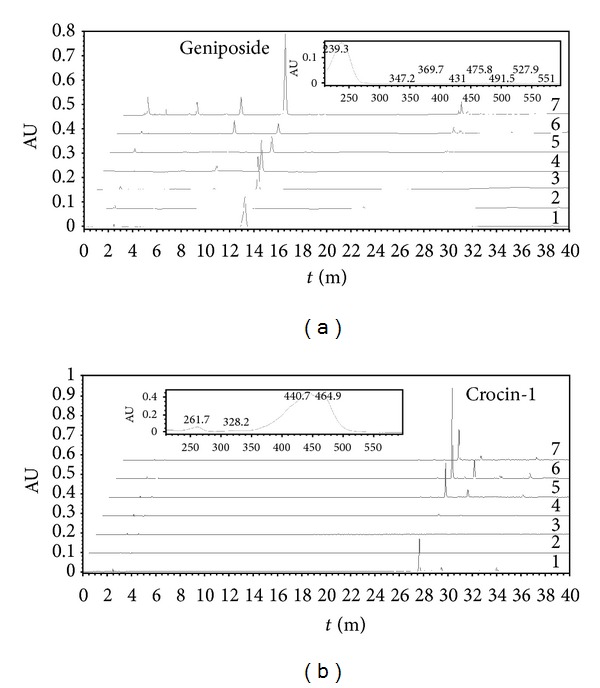
HPLC profile of methanol-water extract from tissues of *G. jasminoides*. The main peak detected corresponds to crocin-1 or geniposide. In (a) and (b), lines: 1, crocin-1 or geniposide; 2, leaves; 3–6, fruits in St I to St IV; 7, authentic gardeniae fruit. The profiles were shifted, intended not to have these major peaks overlapping in the figure. (a) in HPLC results monitored at 238 nm, the inset shows online diode array spectrum of geniposide; (b) in HPLC results monitored at 440 nm, the inset shows online diode array spectrum of crocin-1.

To identify and quantify crocin-1 and geniposide in fruits and leaves, the methanol-water extraction method was used, and the extract was subjected to HPLC separation. During the development of the *G. jasminoides* fruit, the exocarp changes in color from green to red; meanwhile the mesocarp changes in color from colorless to red, the fruits were classified into 4 maturation stages based on the red coloration. The fruits from these four maturation stages were analyzed. The typical HPLC chromatogram of the methanol-water extracts from gardenia leaves and fruits recorded at 238 nm and 440 nm is shown in Figure 2. The peak is identified as crocin-1 and geniposide by comparing its absorption spectrums and retention times with the authentic compounds. 

**Figure 3:**
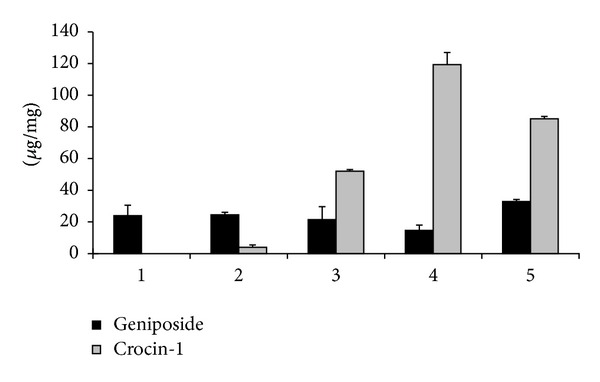
Crocins and geniposide contents of gardenia fruits. 1–4: fruits in St I to St IV; 5: authentic gardeniae fruits. Values are mean ± SD; *n* = 3. The SD was estimated to be below 10%.

It was shown from Figure 2 that the crocin-1 and geniposide were not found in leaves. The concentration of crocin-1 during fruit development is shown in Figure 3; it was revealed that the concentration increased from stage II to stage IV and was not detected in stage I, suggesting that the biosynthesis of crocins was continuous during the maturation of gardenia fruits. From stage II to stage IV, the crocin-1 content increased from 3.96 *μ*g/mg to 119.29 *μ*g/mg. 

The geniposide concentration in gardenias fruits was unchanged from stage I to stage III, between 23.16 and 21.52 *μ*g/mg, and was slightly decreased at stage IV, 17.99 *μ*g/mg (Figure 3). Several studies have dealt with the quantitative differences of crocins and geniposide (and other iridoid compounds such as gardenoside) among different *G. jasminoides* cultivars and during fruit maturation and have different results [26, 27]. Fu found that the content of gardenoside during fruit maturation was higher at early and later maturation stages and was lower at the intermediate stage [26]. Chen indicated that the fruit ripening resulted in statistically increased content of crocins, and the geniposide content of gardenia fruits generally decreased when mature levels increased, it can be explained by the fact of a dilution effect induced by fruit growth [27]. Geniposide and gardenoside contents in different *G. jasminoides* organs were also quantitatively determined; it was found that iridoid content was somewhat lower in the vegetative tissues than in the flowers and fruits [23]. We suggest that geniposide was synthesized early during flowering time and maintained synthesis during fruit development and enlarged; at the final maturation stage, the synthesis of geniposide was reduced while the fruit would not enlarge more. Crocin, crocetin, and geniposide are isoprenoids; they are all derived from common precursor, geranyl diphosphate, and then were synthesized in different pathways. From the crocin-1 and geniposide profiles at the various stages of maturation, we suggest that the regulation mechanisms of these metabolic pathways are different.

### 3.2. Transcriptional Expression of GjPSY

**Figure 4:**
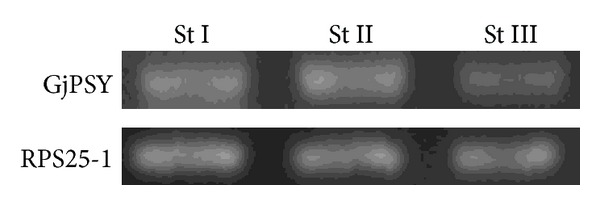
Transcriptional expression of GjPSY gene in gardenia fruits. GjPSY transcript levels in fruits (St I, St II, and St III). The mRNA levels were determined by RT-PCR amplification using specific oligonucleotides for GjPSY. RPS25-1 was used as a control for ubiquitous constitutive gene expression.

Transcriptional expression of GjPSY in gardenia fruits during development stages was analyzed by reverse transcript (RT)-PCR. Fruits in stage I to III were analyzed. Specific oligonucleotides for GjPSY mRNA were designed and used in the expression analysis. The transcript levels of GjPSY were shown to persist in the same abundance in three stages (Figure 4). In fruits, during the crocin-1 accumulation in stage II and stage III, the transcripts level of GjPSY was not increased.

The transcriptional regulation of PSY during fruit maturation has been reported in several plant species [28–30]. In tomato and citrus fruits, high concentrations of carotenoids are accumulated during the fruit maturation; the expression of carotenoid biosynthetic genes during fruit maturation has been studied. In tomato, there are two PSY genes, PSY1 and PSY2, and both PSY1 and PSY2 genes were found to be expressed in seedlings, mature leaves, and fruits of tomato. The transcript level of PSY1 in seedlings and in late stages of fruit ripening is much higher than PSY2, whereas PSY2 transcripts are predominating in mature leaves [29]. GjPSY shows higher sequence similarity to tomato PSY2 than to tomato PSY1, which implies that it is not the fruit ripening-related PSY gene in *G. jasminoides*. In citrus fruits, it was suggested that the carotenoid accumulation during citrus fruit maturation was highly regulated by the coordination of the expression among carotenoid biosynthetic genes; the transcriptional regulation of PSY during fruit maturation is complicated [28]. In this paper, it is the first report on the transcriptional regulation of PSY during *G. jasminoides* fruit maturation.

## 4. Conclusion

In conclusion, (1) concentration of crocin-1 is the highest at a late maturation stage; (2) the concentration of geniposide does not change significantly during maturing, and was slightly decreased at later maturation; (3) the transcriptional expression analysis revealed that the GjPSY is constitutively expressed in fruits at different developmental stages. Therefore, it appears that the transcript level of the GjPSY is not connected with the fruit maturation process.
